# The Effect of Traditional Chinese Exercises on Blood Pressure in Patients with Hypertension: A Systematic Review and Meta-Analysis

**DOI:** 10.1155/2023/2897664

**Published:** 2023-02-09

**Authors:** Jiewei Ma, Lianzhen Ma, Shihan Lu, Yue Sun, Haipeng Bao

**Affiliations:** School of Physical Education and Sports Science, South China Normal University, Guangzhou 510006, China

## Abstract

In the context of the increasing number of patients with hypertension, exercise intervention is an excellent alternative or adjunctive treatment for hypertension. Traditional Chinese exercises are excellent physical and mental exercise methods. Although some studies have reviewed the effects of Chinese traditional exercise on patients with hypertension, most of the reviews only involved a single category of traditional exercise. Furthermore, few studies have conducted in-depth analysis of the combined intervention methods of traditional Chinese exercise, and there are high heterogeneity. This study evaluates the current clinical evidence of Chinese traditional exercises in the treatment of essential hypertension. A total of 49 randomized controlled trials with 4207 hypertensive patients were selected according to the inclusion criteria by searching all relevant studies from the establishment of six electronic databases until September 10, 2022. Among them, 24 used tai chi and 25 used Qigong, including Yijinjing, Wuqinxi, Liuzijue, Baduanjin, and Guolin Qigong. This study divided four subgroups according to the type of intervention to explore the source of heterogeneity among studies and found that traditional Chinese exercises can assist or even may replace traditional treatments. The results of meta-analysis showed that compared with the use of antihypertensive drugs alone or in health education, the addition of Chinese traditional exercises showed significant effects in regulating the systolic blood pressure and diastolic blood pressure in hypertensive patients. Although the results show that traditional Chinese exercise are effective, the clinical evidence will be affected by the low quality of most randomized controlled trials. More rigorously designed trials are needed in the future to further validate it.

## 1. Introduction

Due to the rapid development of human society, people's diet structure and living habits have changed greatly. The number of patients with hypertension has increased rapidly on account of the excessive intake of high-protein and high-fat food, irregular rest, lack of exercise, smoking and drinking, and other bad behaviors. Hypertension is a clinical syndrome in which blood pressure in the blood vessels continuously rises and is considered the “silent killer” [1]. More than a billion people which include more than a quarter of men and a fifth of women are suffering from this disease, and it will be 1.56 billion by 2025, according to the World Health Organization [2]. If the blood pressure is higher than the normal level for a long time, it will lead to myocardial infarction, stroke, coronary heart disease, heart failure, sudden death, and even death if it is not treated in time [3, 4]. At present, there are three main methods to treat hypertension, namely, drug therapy, lifestyle intervention, and device intervention, among which drug therapy is the best way to control the blood pressure level. Now, common antihypertensive drugs include calcium channel blockers (CCB), angiotensin converting enzyme inhibitors (ACEI) and angiotensin receptor blockers (ARB), diuretics, and beta blockers five classes, but long-term use of antihypertensive drugs has side effects, as the growth of the medication time lead to enhanced resistance [5]. In addition, drug treatment imposes a significant financial burden on the poor. However, the treatment of hypertension is often accompanied by the treatment of lifestyle intervention because most of the hypertension patients are caused by poor lifestyle and primary hypertension. Therefore, lifestyle intervention can effectively reduce the high blood pressure level of some hypertensive patients and effectively reduce the financial pressure of patients. Common lifestyle interventions include reducing sodium intake and increasing potassium intake, eating a reasonable diet, controlling weight, no to smoking, limiting alcohol consumption, increasing exercise, and reducing mental stress [4].

As a lifestyle intervention, exercise therapy has been widely used in hospitals. Research shows that regular exercise can reduce cardiovascular death and all risk of death [6]. To lower blood pressure levels, the researchers recommend 30‒60 minutes of moderate-intensity exercise four to seven times a week, in addition to daily activities [7]. The forms of exercise include aerobic exercise, resistance exercise, and stretching exercise [8]. The common traditional Chinese exercises are Taiji, Baduanjin, Yijinjing, Wuqinxi, Liuzijue, and so on. Taiji is basically made up of 24 movements that emphasize gentleness, slowness, relaxation, meditation, and calm breathing; Baduanjin is a Qigong guidance technique composed of eight groups of movements, combined with meditation and regular breathing; Yijinjing is a fitness Qigong composed of twelve movements; Wuqinxi is a fitness Qigong that imitates the activities of tiger, deer, bear, ape, and bird; Liuzijue is a fitness Qigong that affects the movement of different zang-fu organs, meridians, and collaterals, Qi and blood through the different pronunciation of the six words, namely, “Si,” “Hu,” “Xu,” “Chui,” and “Xi” [9, 10]. Most traditional Chinese exercises are mind-body exercises that combine body movements, breathing patterns, and meditation [11]. They can not only exercise people's body but also reduce people's psychological stress and fully improve people's physical and mental health. Recently, they have been widely used as an alternative drug treatment for many chronic diseases, such as Parkinson's disease, stroke, chronic obstructive pulmonary disease, and cancer, and are welcomed by patients with chronic diseases worldwide. For hypertension, Chinese traditional exercise meets the two options of lifestyle intervention to increase exercise and reduce mental stress, which shows that it has the ability to improve the health status of patients with hypertension, and because exercise occupies a small space, it can be exercised at any time in the hospital ward or at home and other places of daily life.

At present, many studies have proved that Tai Chi and Qigong in Chinese traditional exercises can effectively help hypertensive patients to reduce their blood pressure [12, 13] and improve their quality of life [10]. But most of the reviews only cover a single category of Chinese traditional exercises, such as Tai Chi or Baduanjin [14, 15]. Although previous reviews have described the impact of traditional Chinese exercise in hypertensive patients, most of the meta-analyses have high heterogeneity and do not explain the reasons for the high heterogeneity [16], so the reliability of these meta-analysis results is questionable. Moreover, few previous studies have conducted an in-depth analysis of the joint intervention methods of Chinese traditional exercises. Therefore, this study will take Chinese traditional exercises as a whole and systematically review and evaluate the effects of Chinese traditional exercises combined with antihypertensive drugs and health education on the blood pressure level in patients with essential hypertension so as to provide a reference for subsequent researchers.

## 2. Methods

### 2.1. Search Strategy and Data Source

This study searched all relevant trials from six electronic databases including PubMed, Web Of Science, Cochrane, CNKI, VIP, and WANFANG DATA from the beginning to September 10, 2022. The search strategy of each database is shown in Table 1. To ensure a rigorous and thorough study search, two authors independently screened and evaluated all articles retrieved from the database according to the inclusion criteria, and disagreements were discussed and resolved with the third author.

### 2.2. Eligibility Criteria

In accordance with PICOS (participant, intervention, comparison, outcome, study), the eligibility criteria for this systematic review were as follows:

1. Patients with essential hypertension and no restrictions on nationality, gender, or age
2. Must be randomized controlled trials
3. The main intervention measure of experimental groups was Tai Chi or Qigong. Qigong included Wuqinxi, Baduan Jin, Yijinjing, Liuzijue, and other Qigong
4. The intervention measure of control groups included drug therapy, health education, other sports, and no intervention
5. In addition to hypertension, no other serious diseases, such as diabetes, coronary heart disease, apoplexy, and renal failure
6. Blood pressure (systolic and diastolic) was measured

The criteria for exclusion were as follows:

1. No data for extraction
2. Repeated experimental data appeared in several articles
3. The required outcome measures, SBP (systolic blood pressure) and DBP (diastolic blood pressure), were not available
4. Study protocol
5. The full text could not be found or is unavailable

### 2.3. Data Extraction

The data extraction work shall be carried out by two authors, respectively. If there is any disagreement between the two authors in this step, the third author shall solve the problem. In this study, the following data were extracted: the first author of the article, year of publication, sample size, diagnostic criteria for hypertension, age of the participants and blood pressure levels before and after treatment, details of the intervention, and outcome measures. The EndNote 20 reference management tool was used to organize papers and generate citations.

### 2.4. Risk of Bias Assessment

The criteria in the revised Cochrane risk-of-bias tool for randomized trails, RoB2, were used to independently assess the methodological quality of trials [17]. The items included 5 domains of bias: bias arising from the randomization process, bias due to deviations from intended interventions, bias due to missing outcome data, bias in measurement of the outcome, and bias in selection of the reported result.

### 2.5. Statistical Analysis

Review Manager software (RevMan 5.4, Cochrane Collaboration, 2022) will be used to perform the meta-analysis. Analyses were performed using the mean, standard deviation, and number of participants for each study and a random effects model. The chi-square test and *i*-squared statistic were used to assess heterogeneity between studies and were considered significant when *i*-squared was greater than 50%. In addition, if at least 10 trials were included in the meta-analysis, publication bias was assessed using funnel plot asymmetry. When *p* < 0.05, the results will be considered statistically significant.

### 2.6. Subgroup Analysis

To avoid high heterogeneity among the studies, the analyses were divided into four subgroups according to the type of intervention (TCE (traditional Chinese exercise) vs. Nonintervention, TCE + Health Education versus only Health Education. TCE + AHD (antihypertensive drug) versus only AHD, TCE + Health Education + AHD versus Health Education + AHD).

### 2.7. Protocol Registration

This systematic review and meta-analysis were conducted according to the PRISMA 2020 statement: an updated guideline for reporting systematic reviews [18], and the registration number was CRD42022360208.

PROSPERO 2022 CRD42022360208 was available from https://www.crd.york.ac.uk/prospero/display_record.php?ID=CRD42022360208.

## 3. Results

### 3.1. Study Search Result

**Figure 1:**
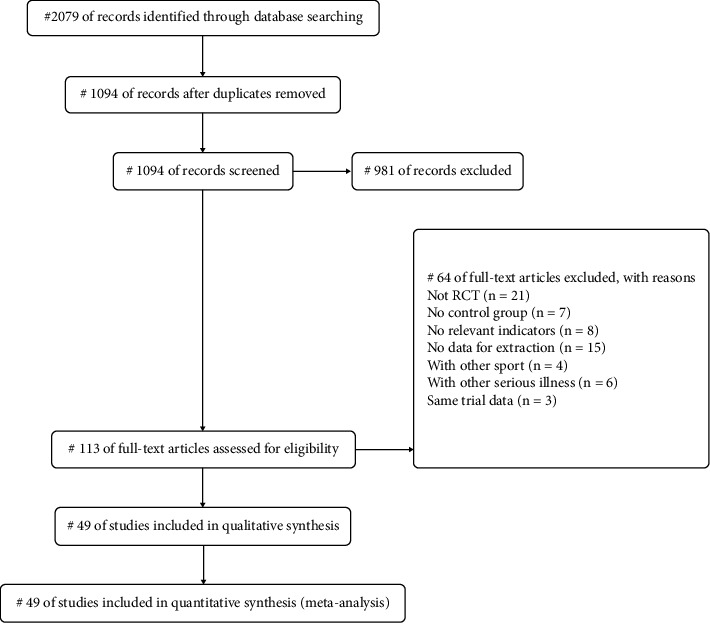
PRISMA flow diagram.

The process of study search and selection is shown in Figure 1. A total of 2079 articles were retrieved from the databases: PubMed (*n* = 115), Web of Science (*n* = 254), Cochrane (*n* = 95), CNKI (*n* = 641), CQVIP (*n* = 359), and WANFANG (*n* = 615). After eliminating duplicate studies, 1094 articles remained. After reading the title and abstract, 981 articles were screened, and 113 articles were included in the full-text screening. Through the entire reading, 64 studies were excluded for the following reasons: nonrandomized controlled trial (*n* = 21), no control group (*n* = 7), no relevant outcome measures (*n* = 8), data could not be extracted (*n* = 15), intervention measures other than traditional Chinese exercise (*n* = 4), patients with other serious diseases (*n* = 6), and similar trial data (*n* = 3). Finally, a total of 49 articles were included in the study.

### 3.2. Characteristics of Included Studies

The basic characteristics of all the included articles are shown in Table 2. All included RCTS were published between 1997 and 2021. The 49 studies involved a total of 4207 patients between the ages of 40 and 80, including 2168 in the intervention group and 2039 in the control group. The traditional Chinese exercise intervention types included in the study were Tai Chi [21, 23, 25, 28, 33, 34, 40, 44, 45, 32, 36, 37, 41, 43–45, 50–57, 59–61, 65, 66], Baduanjin [22, 24, 26, 27, 30, 33, 40, 49, 58, 62, 64, 67, 68], Wuqinxi [39], Yijinjing [69], Liuzijue [63], Mawangdui Qigong [20], and other Qigong [12, 19, 29, 34, 35, 42, 47, 48]. The intervention group included only Chinese traditional exercise, Chinese traditional exercise combined with antihypertensive drugs, Chinese traditional exercise combined with health education, and Chinese traditional exercise combined with health education and antihypertensive drugs. The control group included blank control, walking only, antihypertensive drugs only, health education only, aerobic exercise only, walking combined with antihypertensive drugs, and health education combined with antihypertensive drugs. The drugs involved in the included studies were nifedipine, Norvasc, Telmisartan, Amlodipine Besylate, Calcium Channel Blocker, and Angiotensin-Converting Enzyme Inhibitor, Angiotensin Receptor Blocker, Diuretic, Amlodipine, Cilazpril, Thiazide Diuretic, and Tian Ma Gou Teng Yin. The duration of the included interventions ranged from 6 weeks to 24 months, with sessions ranging from 15 to 75 minutes and frequency of exercise ranging from 2 to 14 times per week. In 49 studies, blood pressure was used as the outcome index.

### 3.3. Risk of Bias

**Figure 2:**
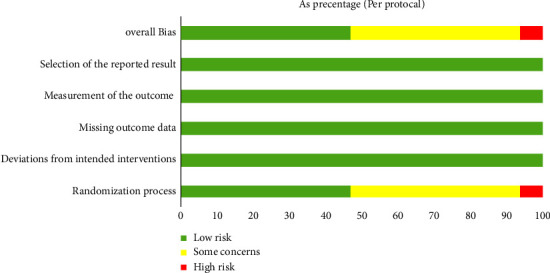
Risk of bias graph.

**Figure 3:**
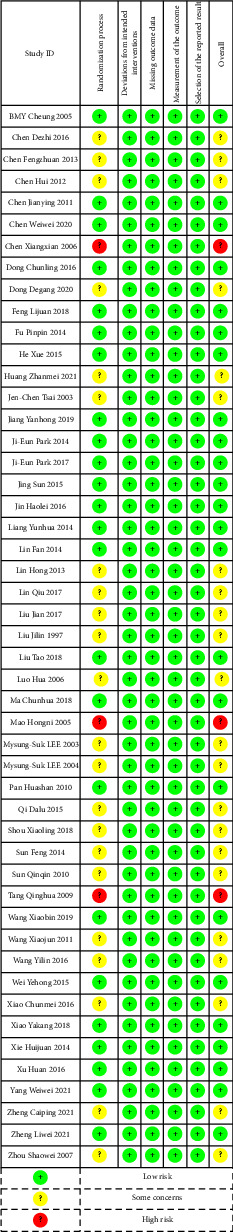
Risk of bias summary.

Figures 2 and 3 show the quality assessment of the included RCTS. All trials mentioned random assignment of subjects, but only 23 trials clearly explained the process of random sequence generation, and none of the others mentioned detailed randomization methods. For allocation concealment, only 8 studies used opaque envelopes, computer random assignment, etc., to conceal allocation from researchers and subjects, 14 studies used the random number table method for allocation, and 3 trials were grouped according to age, test data, etc. Other studies did not explicitly describe allocation concealment. In addition, only two studies blinded subjects and participants, three trials mentioned no blinding, and none of the others explicitly described whether blinding was performed. Only four studies blinded the outcome assessors. But the assessors felt that exercise therapy was not truly double-blind and that the outcome assessors had little impact on the results. In conclusion, more than half of the literature was at a risk of quality, and three of them were at a high risk.

### 3.4. Study Results

All included trials [12–18, 21, 23, 25, 28, 36, 41, 43–45, 54, 55, 57, 61, 66] and [20, 22, 24, 26, 27, 30–33, 37–39, 49–53, 56, 58–60, 62–65, 67–69] compared the effects of traditional Chinese exercise therapy alone or in combination with antihypertensive drugs on blood pressure levels in patients with essential hypertension. All randomized controlled trials reported changes in blood pressure levels, and the results of the meta-analysis are shown in Table 3.

### 3.5. TCE Intervention for Hypertension

**Figure 4:**
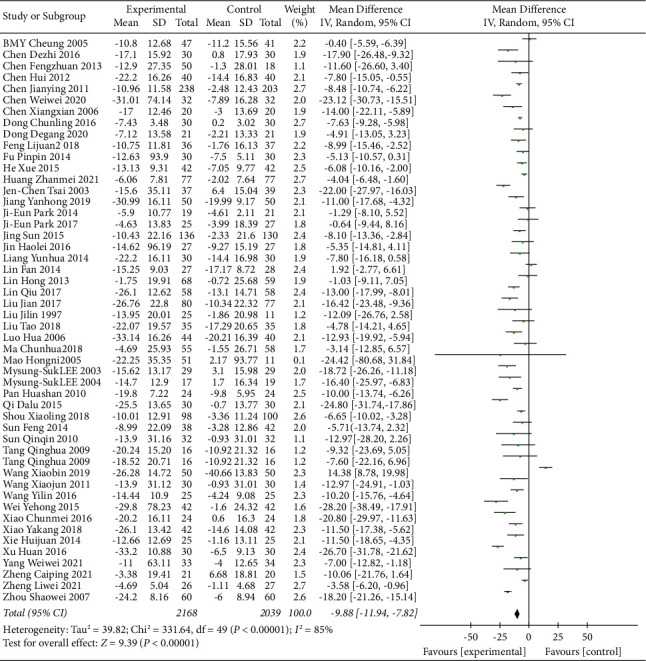
Meta-analysis of the effects of traditional Chinese exercises on systolic blood pressure.

**Figure 5:**
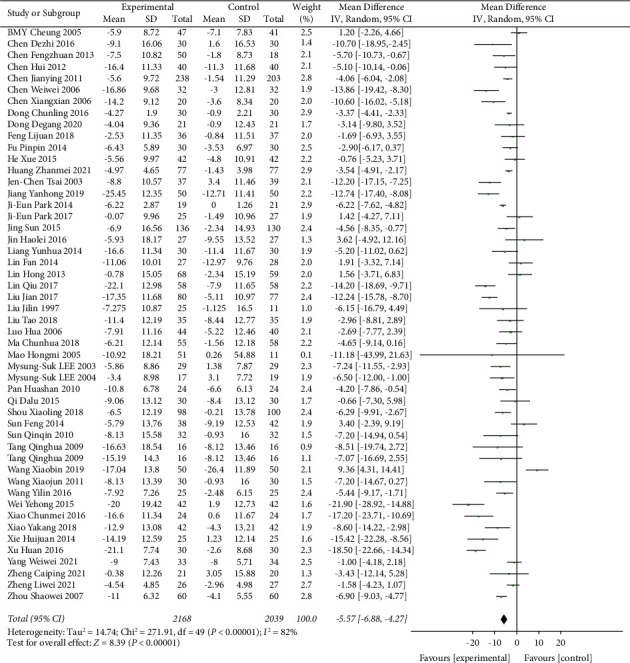
Meta-analysis of the effects of traditional Chinese exercises on diastolic blood pressure.

A total of 4207 participants were included in 49 trials of TCE intervention to explore the effect of traditional Chinese exercise on blood pressure in hypertensive patients. It can be seen from Figures 4 and 5 that SBP of hypertensive patients was significantly reduced in the Chinese traditional exercise group compared with the control group (MD = −9.88, 95% CI: [−11.94, −7.82], *p* < 0.00001, and *I*^2^ = 85%). There was also a significant reduction in DBP in hypertensive patients (MD = −5.57, 95% CI: [−6.88, −4.27], *p* < 0.00001, and *I*^2^ = 82%). But due to high heterogeneity between studies in the following paragraphs depending on the type of intervention is divided into four subgroups to analyze the source of heterogeneity among studies.

### 3.6. TCE versus Nonintervention for Hypertension

**Figure 6:**
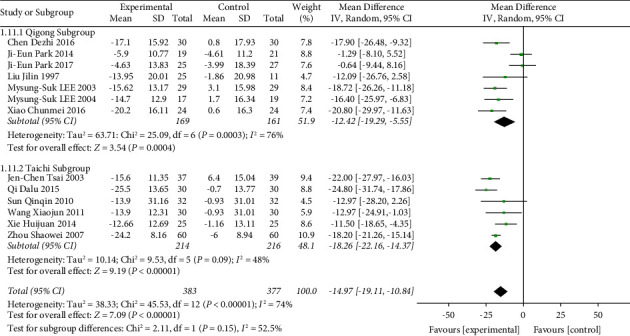
Meta-analysis of the effects of traditional Chinese exercises on systolic blood pressure versus nonintervention.

**Figure 7:**
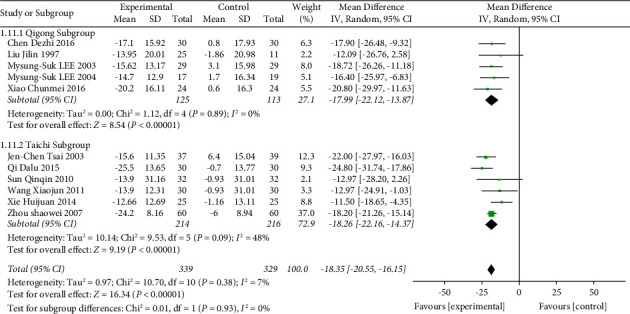
Meta-analysis of the effects of traditional Chinese exercises on systolic blood pressure versus nonintervention (deleted [39, 58]).

**Figure 8:**
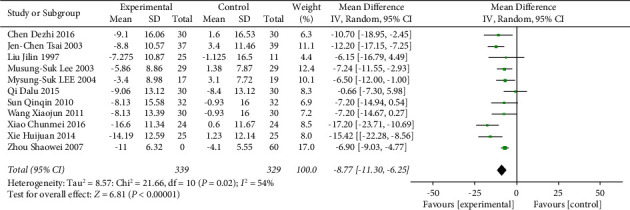
Meta-analysis of the effects of traditional Chinese exercises on diastolic blood pressure versus nonintervention.

The TCE vs. nonintervention subgroup included 13 trials with a total of 760 participants. Compared with the nonintervention group, the Chinese traditional exercise group significantly reduced the SBP of hypertensive patients (MD = −14.97, 95% CI: [−19.11, −10.84], *p* < 0.00001, *I*^2^ = 74%), as shown in Figure 6. Although there was still significant heterogeneity among the studies, it was reduced compared with Figure 4. To further explore the source of heterogeneity, this subgroup was divided into the Qigong group (MD = −12.42, 95% CI: [−19.29, −5.55], *p* < 0.00001, and *I*^2^ = 76%) and Tai Chi group (MD = −18.26, 95% CI: [−22.16, −14.37], *p* < 0.00001, and *I*^2^ = 48%). It can be seen that after the classification of traditional Chinese sports, the heterogeneity of the Tai Chi group was significantly reduced, but the heterogeneity of the Qigong group was higher. It was preliminarily determined that the heterogeneity was mainly from the Qigong group. A review of data from each study revealed that two studies [39, 58] were designed only for patients with prehypertension, and heterogeneity was significantly reduced after deletion (MD = −18.35, 95% CI: [−20.55, −16.15], *p* < 0.00001, and *I*^2^ = 7%), as shown in Figure 7. Compared with the nonintervention group, the Chinese traditional exercise group significantly reduced the DBP of hypertension patients (MD = −8.77, 95% CI: [−11.30, −6.25], *p* < 0.00001, and *I*^2^ = 54%), as shown in Figure 8, and there was still a large heterogeneity among studies, but it was significantly reduced compared with Figure 5, which was in an acceptable range, so the random effect model was used.

### 3.7. TCE + HE versus HE for Hypertension

**Figure 9:**
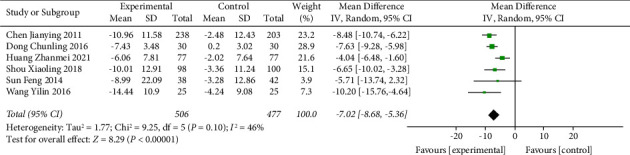
Meta-analysis of the effects of traditional Chinese exercises and health education on systolic blood pressure versus health education.

**Figure 10:**
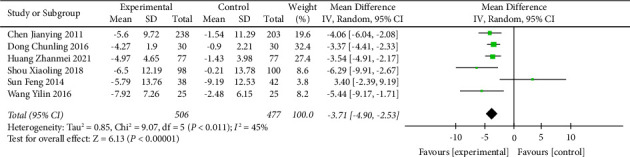
Meta-analysis of the effects of traditional Chinese exercises and health education on diastolic blood pressure versus health education.

The TCE + HE vs. HE subgroup was included in 6 trials, with a total of 983 participants. Compared with the health education group only, the Chinese traditional exercise combined with the health education group had a significant effect on reducing the SBP level of hypertensive patients (MD = −7.02, 95% CI: [−8.68, −5.36], *p* < 0.00001, and *I*^2^ = 46%), as shown in Figure 9. In addition, this subgroup also had a significant effect on reducing the DBP level in hypertensive patients (MD = −3.71, 95% CI: [−4.90, −2.53], *p* < 0.00001, and *I*^2^ = 45%), as shown in Figure 10. When analyzed in this subgroup, heterogeneity was significantly reduced compared with the intervention in the overall TCE group.

### 3.8. TCE + AHD versus AHD for Hypertension

**Figure 11:**
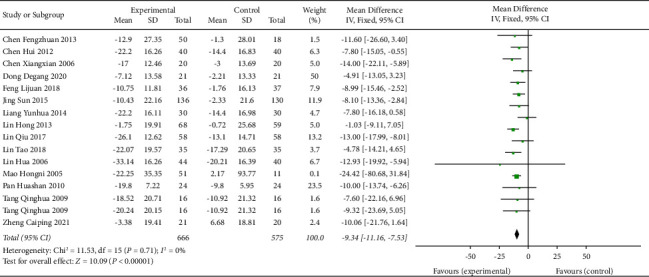
Meta-analysis of the effects of traditional Chinese exercises and antihypertension drugs on systolic blood pressure versus antihypertension drugs.

**Figure 12:**
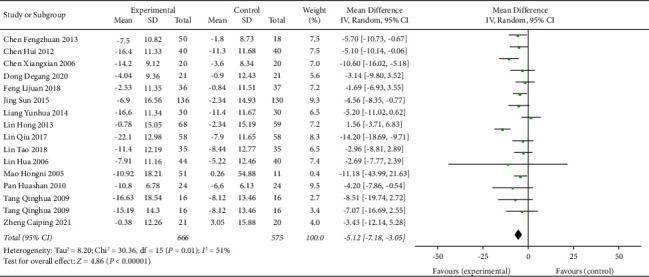
Meta-analysis of the effects of traditional Chinese exercises and antihypertension drugs on diastolic blood pressure versus antihypertension drugs.

16 trials with 1241 participants were included in the TCE + AHD vs. AHD subgroup. Compared with the antihypertensive drug group, the Chinese traditional exercise combined with the antihypertensive drug group had a significant effect on reducing the SBP level in hypertensive patients (MD = −9.34, 95% CI: [−11.16, −7.53], *p* < 0.00001, and *I*^2^ = 0%), and the heterogeneity was significantly lower than that of the overall TCE group intervention because for *I*^2^ = 0, the fixed effects model was used, as shown in Figure 11. Chinese traditional exercise combined with antihypertensive drugs has a significant effect on reducing the DBP level in hypertensive patients (MD = −5.12, 95% CI: [−7.18, −3.05], *p* < 0.00001, and *I*^2^ = 51%), and heterogeneity was significantly reduced compared with the overall TCE group intervention, as shown in Figure 12 using the random effects model.

### 3.9. TCE + AHD + HE versus AHD + HE for Hypertension

**Figure 13:**
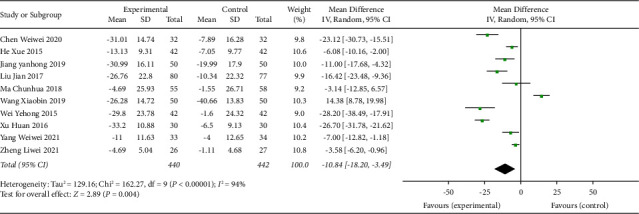
Meta-analysis of the effects of traditional Chinese exercises and antihypertension drugs and health education on systolic blood pressure versus antihypertension drugs and health education.

**Figure 14:**
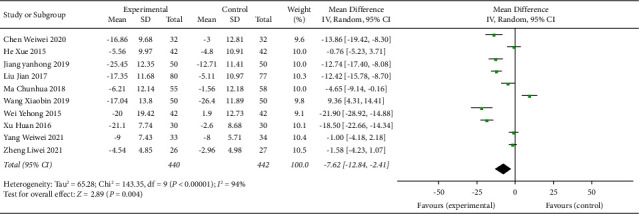
Meta-analysis of the effects of traditional Chinese exercises and antihypertension drugs and health education on diastolic blood pressure versus antihypertension drugs and health education.

The TCE + AHD + HE vs. AHD + HE subgroup was included in 10 trials with 840 participants. The Chinese traditional exercise combined with antihypertensive drugs and the health education group had a significant effect on reducing the SBP level of hypertensive patients (MD = −10.84, 95% CI: [−18.20, −3.49], *p* < 0.004, and *I*^2^ = 94%), also had a significant effect on reducing the DBP level in hypertensive patients (MD = −7.62, 95% CI: [−12.84, −2.41], *p* < 0.004, and *I*^2^ = 94%), and heterogeneity was increased compared with the overall TCE group intervention, as shown in Figures 13 and 14. Since this subgroup included both traditional Chinese exercise therapy and drug therapy and health education, researchers tried to further analyze its high heterogeneity, such as the traditional exercise type and drug type, but still could not solve the problem of high heterogeneity. The researchers hypothesized that some studies did not clarify the type of drug used or that there was high heterogeneity due to the intersection of multiple interventions (traditional Chinese exercise, antihypertensive drugs, and health education).

### 3.10. Publication Bias

**Figure 15:**
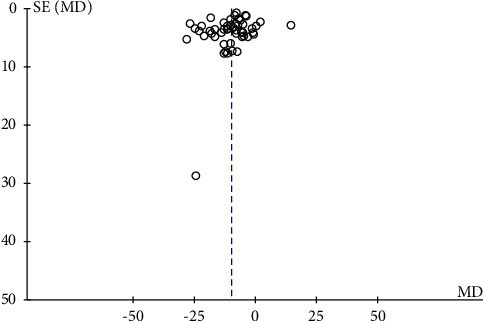
Funnel plot of 49 randomized control trials on the effect of traditional Chinese exercises on hypertension.

Publication bias in 49 RCTS was assessed using funnel plots, and as shown in Figure 15, publication bias in the studies was small.

## 4. Discussion

Scientific evidence has proved that exercise training is effective in treating hypertension. However, for hypertension patients with poor physical condition, there is a certain risk of moderate and high intensity exercise. Most traditional Chinese exercises are mostly physical and mental exercise with the main purpose of health preservation, which has been widely used in the treatment of various chronic diseases. This study systematically reviewed the previous literature with an objective assessment of the effect of TCE on blood pressure levels in hypertensive patients to find an appropriate treatment.

### 4.1. Summary of Research Results

According to the study inclusion criteria, 49 randomized controlled trials with a total of 4207 hypertensive patients were selected. The study found that the frequency of traditional exercise practice was 5–7 times per week, and each exercise time was 30–60 minutes, which was the most commonly used test setting in the included studies. According to the results of the meta-analysis, the antihypertensive effect was the most obvious in hypertensive patients undergoing Chinese traditional exercise when compared with the nonintervention group. Secondly, in the TCE + AHD + HE group, under the combination of the treatment of the three intervention methods, it also had a very significant effect on the reduction of blood pressure. The third is the TCE + AHD group, and compared with AHD, it also played a significant antihypertensive effect. Finally, we have the TCE + HE group, and because there is no drug intervention involved, the antihypertensive effect is not as good as the previous groups, but it is also an excellent option for people who cannot take drugs. Therefore, Chinese traditional exercise can effectively reduce blood pressure levels in hypertensive patients and assist drugs and health education treatment to promote the recovery of patients with hypertension.

### 4.2. Advantages and Limitations

Since Chinese traditional exercises are physical and mental sports with the main purpose of health preservation, the difference between this study and previous studies is that Chinese traditional exercises are regarded as a whole combined with medicine and health education therapy, rather than individual Tai Chi or Baduanjin alone. The studies included in this paper showed great heterogeneity firstly, but they had a significant effect in reducing the blood pressure level, which was statistically significant, and proved that Chinese traditional exercises are superior to the control group in improving the blood pressure level in hypertensive patients. In view of the high interstudy heterogeneity, the study was divided into four subgroups (TCE versus nonintervention group, TCE + health education versus pure health education group, TCE + AHD versus pure AHD group, and TCE + health education + AHD versus health education + AHD group) to explore the source of interstudy heterogeneity. After further subgroup analysis, the heterogeneity was significantly reduced in the TCE vs. nonintervention group, TCE + health education vs. only health education group, and TCE + AHD vs. only AHD group. However, for the TCE + health education + AHD versus health education + AHD group, the heterogeneity did not decrease but increased. Preliminary speculation suggested that the heterogeneity in each study could not be reduced due to differences among multiple interventions, such as the types of antihypertensive drugs, the content and effect of health education, and so on. Therefore, the adjuvant therapy of Chinese traditional exercise can lower the blood pressure level in hypertensive patients.

Although this meta-analysis found a positive effect of the Chinese traditional exercise on the treatment of hypertensive patients, the clinical evidence of their ability to treat essential hypertension may be weakened by the low methodological quality of most included studies. First, most RCT trials only mentioned randomization but did not explain in detail the method and process of randomization. Secondly, most of the experiments did not mention the hidden problem of allocation, and some experiments used random number table allocation, which has certain risks. All these issues contributed to the risk of bias in this study.

## 5. Conclusion

From the results of the present study, we can draw the following conclusions. Compared with the use of antihypertensive drugs alone or in health education, the addition of Chinese traditional exercises showed significant effects in regulating the SBP and DBP levels in hypertensive patients. In addition, the study found that for the treatment of hypertension, most of the Chinese traditional exercises are Taijiquan and Baduanjin, while other types of sports such as Wuqinxi, Liuzijue, Yijinjing, and other tests are rare. Moreover, according to the analysis of reduced subgroup heterogeneity, it is suggested that future exercise prescription formulation can be TCE vs. nonintervention, TCE + health education vs. health education, and intervention prescription design of TCE + AHD vs. AHD, which can better reflect the effect of traditional exercise on hypertensive patients.

In summary, although some evidence can prove that Chinese traditional exercise can have a positive effect on the blood pressure level in hypertensive patients, the evidence is still weak due to the insufficient number of included studies and methodological quality issues. Therefore, it is hoped that more rigorously designed randomized controlled trials with more types of traditional exercise will emerge to confirm the evidence of traditional exercise on blood pressure levels in Chinese patients with hypertension.

## Figures and Tables

**Table 1:** The search strategy of databases.

Databases	Search strategy
PubMed	((Tai chi[Title/Abstract]) OR (Tai ji [Title/Abstract]) OR (Tai-ji [Title/Abstract]) OR (Baduanjin [Title/Abstract]) OR (Wuqinxi [Title/Abstract]) OR (Liuzijue [Title/Abstract]) OR (Yijinjing [Title/Abstract]) OR (Qigong [Title/Abstract])) AND (Hypertension [Title/Abstract])
Web of science	((TS = (qigong)) OR TS = (Tai chi) OR TS = (Taiji) OR TS = (Tai-ji) OR TS = (Baduanjin) OR TS = (Wuqinxi) OR TS = (Liuzijue) OR TS = (Yijinjing)) AND TS = (Hypertension)
Cochrane	((“Tai Chi”): ti, ab, kw OR (“Taiji”): ti, ab, kw OR (“Tai-ji”): ti, ab, kw OR (“Buduanjin”): ti, ab, kw OR (“Wuqinxi”): ti, ab, kw OR (“Liuzijue”): ti, ab, kw OR (“qigong”): ti, ab, kw OR (“yijinjing”): ti, ab, kw) and (“Hypertension”): ti, ab, kw
CNKI	SU% (“Taijiquan” + “Baduanjin” + “Wuqinxi” + “Liuzijue” + “Yijinjing” + “Qigong”) and SU = “Gaoxueya”
CQVIP	M = (Qigong OR Yijinjing OR Wuqinxi OR Liuzijue OR Baduanjin OR Taiji) AND M = Gaoxueya
WANFANG DATA	Title or keyword: (Qigong or Taiji or Yijinjing or Wuqinxi or Liuzijue or Baduanjin) and title or keyword: (Gaoxueya)

**Table 2:** The basic characteristics of included studies.

Study	Example (person)		Average age (year)		Intervention		Blood pressure				Exercise prescription			Drug
	Experimental group	Control group	Experimental group	Control group	Experimental group	Control group	Experimental group		Control group		Frequency (weekly)	Time (min)	Duration	
							SBP	DBP	SBP	DBP				
Cheung et al. [19]	47	41	57.2 ± 9.5	51.2 ± 7.4	Guolin qigong	Walking	146.3 ± 7.8	93 ± 4.1	140.9 ± 10.9	93.1 ± 3.5	7	75	16 weeks	
Chen [20]	30	30	NR	NR	Mawangdui qigong	N	155.3 ± 12.7	103.2 ± 13.5	154.6 ± 11.8	102.7 ± 10.7	5	44	6 months	
Chen and Lu [21]	50	18	NR	NR	Tai Chi + AHD	AHD	169.5 ± 21.1	93.5 ± 7.9	170.9 ± 20.4	94.9 ± 6.7	6	30	12 weeks	NR
Chen and Zhou [22]	40	40	59 ± 6	60 ± 5	Baduanjin + AHD	Walking + AHD	158.5 ± 12.5	101.6 ± 8.4	160.2 ± 11.6	100.8 ± 9.2	14	20	24 weeks	Nifedipine
Chen et al. [23]	238	203	NR	NR	Tai Chi + HE	HE	141.91 ± 9.42	82.26 ± 7.87	141.77 ± 8.91	81.7 ± 7.98	5	60	24 months	
Chen et al. [24]	32	32	68.04 ± 9.69	65.71 ± 8.71	Baduanjin + AHD + HE	AHD + HE	166.76 ± 10.86	97.18 ± 6.86	167.43 ± 14.61	96.32 ± 9.18	5	30	6 months	NR
Chen and Lu [25]	20	20	64.3	60.7	Taichi + AHD	AHD	150.4 ± 7.7	97.8 ± 6.5	151.6 ± 8.9	99.7 ± 5.1	7	40	10 weeks	Nifedipine
Dong and Zhang [26]	30	30	NR	NR	Baduanjin + HE	HE	148.73 ± 3.13	94.87 ± 1.66	148.27 ± 2.16	95.27 ± 1.57	14	15–20	2 months	
Dong et al. [27]	21	21	NR	NR	Baduanjin + AHD	AHD	137.25 ± 10.13	92.75 ± 6.4	138.51 ± 8.12	92.52 ± 6.83	5	60	4 months	Norvasc\Telmisartan\Amlodipine Besylate
Feng et al. [28]	36	37	66.33 ± 4.74	67.51 ± 4.09	Tai Chi + AHD	Walking + AHD	144.17 ± 8.96	81.39 ± 8.67	145.22 ± 11.31	81.89 ± 8.88	3	60	12 weeks	NR
Fu [29]	30	30	57.93 ± 6.63	59.53 ± 7.46	Qigong	AHD	134.93 ± 7.08	78.16 ± 4.13	136.3 ± 7.88	80.13 ± 4.48	6	40	3 months	Calcium channel blocker\ angiotensin-converting enzyme inhibitor\angiotensin receptor Blocker\Diuretic
He [30]	42	42	68.51 ± 2.97	68.24 ± 2.45	Baduanjin + AHD + HE	AHD + HE	140.67 ± 6.45	81.23 ± 7.72	139.87 ± 7.04	81.77 ± 8.02	5	30	3 months	NR
Huang Zhanmei [31]	77	77	NR	NR	Baduanjin + HE	HE	147.32 ± 5.35	93.98 ± 2.73	148.34 ± 5.32	94.14 ± 2.7	10	30	3 months	
Tsai [32]	37	39	51.6 ± 16.3	50.5 ± 9.8	Taichi	*N*	142.4 ± 8.6	87.4 ± 8.7	148.2 ± 8.8	86.2 ± 8.4	3	50	12 weeks	
Jiang et al. [33]	50	50	64.67 ± 3.15	65.23 ± 3.23	Baduanjin + AHD + HE	AHD + HE	155.67 ± 12.37	95.68 ± 9.69	159.87 ± 13.69	95.88 ± 10.17	10	30	12 weeks	Amlodipine
Park et al. [34]	19	21	52 ± 4.87	53.85 ± 4.49	Qigong	*N*	134.14 ± 9.63	89 ± 0.894	140.48 ± 10.02	89 ± 0.894	5	30	8 weeks	
Park et al. [35]	25	27	54.52 ± 6.96	52.93 ± 8.45	Qigong	*N*	134.45 ± 10.41	85.23 ± 6.43	130.47 ± 13.93	85.41 ± 7.83	5	50	12 weeks	
Jing and Buys [36]	136	130	NR	NR	Tai Chi + AHD	AHD	130.71 ± 16.65	82.21 ± 7.94	130.46 ± 15.97	81.92 ± 8.25	5 h/week		12 months	NR
Jin and Pang [37]	27	27	NR	NR	Tai Chi	AHD	152.44 ± 16.21	92.92 ± 14.35	150.24 ± 11.36	97.19 ± 9.35	7	40	6 weeks	Amlodipine
Liang et al. [27]	30	30	54.8 ± 7.6	55.7 ± 8.8	Baduanjin + AHD	Walking + AHD	158.6 ± 12.3	101.7 ± 8.5	160.1 ± 11.7	100.9 ± 9.1	10	20	6 months	NR
Lin Fan [38]	27	28	61.26 ± 3.74	62.03 ± 3.51	Baduanjin	AHD	147.93 ± 6.6	88.56 ± 7.65	148.8 ± 6.65	88.57 ± 8.21	14	30	12 weeks	Amlodipine Besylate
Lin and Huang [39]	68	59	NR	NR	Wuqinxi + AHD	AHD	132.13 ± 13.8	78.87 ± 11.04	133.12 ± 18.32	78.37 ± 11.15	6	30	3 months	NR
Qiu and Yan [40]	58	58	NR	NR	Baduanjin + AHD	AHD	149.3 ± 12.5	97.8 ± 12.6	148.7 ± 13.2	96.4 ± 10.7	5	30	6 months	Amlodipine Besylate
Liu [41]	80	77	43 ± 6.57	42.6 ± 5.67	Tai Chi + AHD + HE	AHD + HE	158.76 ± 19.24	98.37 ± 10.01	157.59 ± 18.65	98.35 ± 9.26	14	40	24 weeks	Nifedipine
Liu et al. [42]	25	11	NR	NR	Qigong	*N*	159.3 ± 14.7	90.075 ± 7.8	159.975 ± 13.95	91.875 ± 13.725	5-6	60	10 weeks	
Liu et al. [43]	35	35	62.4 ± 2.4	63.1 ± 2.1	Tai Chi + AHD	AHD	157.96 ± 15.24	97.24 ± 6.58	158.45 ± 15.73	97.85 ± 6.58	7	40–60	6 months	Cilazpril
Luo [44]	44	40	44.74 ± 12.1	44.86 ± 13.05	Tai Chi + AHD	AHD	163.24 ± 12.56	98.78 ± 8.37	161.17 ± 11.93	99.87 ± 94.65	7	45	6 months	Cilazpril
Ma et al. [45]	55	58	70.24 ± 10.25	69.71 ± 10.84	Tai Chi + AHD + HE	AHD + HE	149.06 ± 19.51	90.74 ± 8.24	150.19 ± 18.3	89.16 ± 9.37	3–5	60	24 weeks	NR
Mao Hongni [46]	51	11	45–70	52–72	Tai Chi + AHD	AHD	162.74 ± 26.36	93.66 ± 13.72	161.72 ± 64.96	94.09 ± 38.71	6	60	8 weeks	NR
Lee et al. [47]	29	29	55.8 ± 5.3	61.6 ± 6.6	Qigong	*N*	146.96 ± 9.57	92.75 ± 5.27	148.62 ± 12.16	95.51 ± 5.72	7	30	10 weeks	
Lee et al. [48]	17	19	52.6 ± 5.1	54.3 ± 5.5	Qigong	*N*	152 ± 10.5	97.2 ± 6.5	150 ± 11.8	93.8 ± 6.2	2	30	8 weeks	
Pan and Feng [49]	24	24	62.1 ± 5.8	61.4 ± 7.1	Baduanjin + AHD	AHD	145.2 ± 3.7	94.5 ± 4.1	144.6 ± 3.9	95.3 ± 3.4	10	45	24 weeks	Thiazide Diuretic\Tian Ma Gou Teng Yin
Qi et al. [50]	30	30	59.73 ± 4.35	60.68 ± 8.06	Tai Chi	*N*	151.26 ± 10.53	87.65 ± 10.42	152.01 ± 9.78	86.99 ± 10.42	5	60	12 weeks	
Shou et al. [51]	98	100	52.35 ± 3.26	51.35 ± 4.21	Tai Chi	HE	140.47 ± 8.31	88.79 ± 9.35	141.9 ± 7.93	87.41 ± 9.72	7–14	45–60	3 months	
Sun and Sun [52]	38	42	68.16 ± 4.43	69.10 ± 4.28	Tai Chi	HE	142.79 ± 12.21	86.42 ± 8.56	147.52 ± 6.25	90.24 ± 8.5	7	60	8 weeks	
Sun [53]	32	32	57.19 ± 8.09	57.25 ± 5.63	Tai Chi	N	138.59 ± 24.86	85.47 ± 12.01	141.09 ± 23.44	85.31 ± 11.64	6	60	3 months	
Tang [54]	16	16	63.65 ± 8.71	62.79 ± 7.43	Tai Chi + AHD	AHD	159.37 ± 15.83	99.21 ± 10.55	158.63 ± 16.16	98.44 ± 9.87	3	30	6 months	NR
Wang and Ye [55]	50	50	67.6 ± 4.5	67.4 ± 4.2	Tai Chi + AHD + HE	AHD + HE	160.74 ± 11.3	93.28 ± 9.26	163.53 ± 10.26	94.84 ± 8.35	3	40–60	3 months	NR
Wang et al. [56]	30	30	NR	NR	Tai Chi	*N*	138.59 ± 10.86	85.47 ± 12.01	141.09 ± 23.44	85.31 ± 11.64	5	60	16 weeks	
Wang [44]	25	25	NR	NR	Shaolin Yijinjing + HE	HE	134.4 ± 8.87	88.32 ± 5.99	131.88 ± 6.61	89.56 ± 4.88	7	90	12 weeks	
Yehong et al. [57]	42	42	72 ± 5.56	70 ± 6.08	Tai Chi + AHD + HE	AHD + HE	151.3 ± 21.8	91 ± 16	144.3 ± 19.6	85.6 ± 10.7	7	30–45	12 months	NR
Xiao et al. [58]	24	24	NR	NR	Baduanjin	*N*	156.6 ± 12.3	101.7 ± 8.5	155.1 ± 10.7	100.9 ± 9.1	5	40	6 months	
Xiao [59]	42	42	60.2 ± 4.6	60.5 ± 4.9	Tai Chi	Aerobic exercise	151.4 ± 10.3	90.8 ± 10.5	151.8 ± 10.2	90.4 ± 10.2	5	60	3 months	
Xie and Bai [60]	25	25	NR	NR	Tai Chi	*N*	154.13 ± 7.69	97.78 ± 8.06	153.55 ± 9.8	97 ± 9.6	5	60	12 weeks	
Xu [61]	30	30	38.07 ± 8.09	37.63 ± 9.09	Tai Chi + AHD + HE	AHD + HE	158.4 ± 8.8	97.5 ± 4.2	159.1 ± 7.5	96.7 ± 5	14	10	8 weeks	NR
Yang and Zheng [62]	33	34	63.64 ± 2.47	62.76 ± 1.92	Baduanjin + AHD + HE	AHD + HE	150 ± 10.5	100 ± 7	148 ± 12	101 ± 5.25	5	30	12 weeks	Lercanidipine\Losartan potassium
Zheng et al. [63]	21	20	65.19 ± 6.47	60.70 ± 8.01	Liuzijue + AHD	AHD + HE	142.57 ± 16.18	82.52 ± 9.12	130.6 ± 14.75	84.55 ± 11.9	3	60	12 weeks	NR
Zheng et al. [64]	26	27	NR	NR	Baduanjin + AHD + HE	AHD + HE	134.92 ± 3.76	80.54 ± 4.06	134.44 ± 4.05	80.89 ± 4.34	5	30	24 weeks	Lercanidipine
Zhou [65]	60	60	52.3 ± 10.7	53.4 ± 11.2	Tai Chi	*N*	147.8 ± 5.3	93.2 ± 2.5	148.3 ± 5.8	92.7 ± 2.6	7	60	12 weeks	

NR: not reported; N: nonintervention; AHD: antihypertensive drugs; HE: health education.

**Table 3:** The results of the meta-analysis.

Groups	Outcomes	Participants	Mean difference IV, random, 95% CI	Heterogeneity (%)	*p* value
TCE	SBP	4207	−9.88 [−11.94, −7.82]	*I* ^2^ = 85	*p* < 0.00001
	DBP		−5.57 [−6.88, −4.27]	*I* ^2^ = 82	*p* < 0.00001

TCE versus nonintervention	SBP	668	−18.35 [−20.55, −16.15]	*I* ^2^ = 7	*p* < 0.00001
	DBP		−8.77 [−11.30, −6.25]	*I* ^2^ = 54	*p* < 0.00001

TCE + HE versus HE	SBP	983	−7.02 [−8.68, −5.36]	*I* ^2^ = 46	*p* < 0.00001
	DBP		−3.71 [−4.90, −2.53]	*I* ^2^ = 45	*p* < 0.00001

TCE + AHD versus AHD	SBP	1241	−9.34 [−11.16, −7.53]	*I* ^2^ = 0	*p* < 0.00001
	DBP		−5.12 [−7.18, −3.05]	*I* ^2^ = 51	*p* < 0.00001

TCE + AHD + HE versus AHD + HE	SBP	882	−10.84 [−18.20, −3.49]	*I* ^2^ = 94	*p* < 0.004
	DBP		−7.62 [−12.84, −2.41]	*I* ^2^ = 94	*p* < 0.004

## Data Availability

The data used to support the findings of this study are included within the article and can be made available upon request to the corresponding author.
